# Mechanistic Insights Into the Anticancer Properties of the Auranofin Analog Au(PEt_3_)I: A Theoretical and Experimental Study

**DOI:** 10.3389/fchem.2020.00812

**Published:** 2020-09-18

**Authors:** Iogann Tolbatov, Damiano Cirri, Lorella Marchetti, Alessandro Marrone, Cecilia Coletti, Nazzareno Re, Diego La Mendola, Luigi Messori, Tiziano Marzo, Chiara Gabbiani, Alessandro Pratesi

**Affiliations:** ^1^Department of Pharmacy, University “G. D'Annunzio” Chieti-Pescara, Chieti, Italy; ^2^Department of Chemistry and Industrial Chemistry (DCCI), University of Pisa, Pisa, Italy; ^3^Department of Pharmacy, University of Pisa, Pisa, Italy; ^4^Laboratory of Metals in Medicine (MetMed), Department of Chemistry “U. Schiff”, University of Florence, Florence, Italy; ^5^CISUP-Centro per l'Integrazione della Strumentazione Scientifica dell'Università di Pisa, University of Pisa, Pisa, Italy

**Keywords:** cancer, ESI-MS, DFT, auranofin, gold, metal-based anticancer drugs, protein metalation, ^31^PNMR

## Abstract

Au(PEt_3_)I (AF-I hereafter), the iodide analog of the FDA-approved drug auranofin (AF hereafter), is a promising anticancer agent that produces its pharmacological effects through interaction with non-genomic targets such as the thioredoxin reductase system. AF-I is endowed with a very favorable biochemical profile showing potent *in vitro* cytotoxic activity against several cancer types including ovarian and colorectal cancer. Remarkably, in a recent publication, some of us reported that AF-I induces an almost complete and rapid remission in an orthotopic *in vivo* mouse model of ovarian cancer. The cytotoxic potency does not bring about highly severe side effects, making AF-I very well-tolerated even for higher doses, even more so than the pharmacologically active ones. All these promising features led us to expand our studies on the mechanistic aspects underlying the antitumor activity of AF-I. We report here on an integrated experimental and theoretical study on the reactivity of AF-I, in comparison with auranofin, toward relevant aminoacidic residues or their molecular models. Results point out that the replacement of the thiosugar moiety with iodide significantly affects the overall reactivity toward the amino acid residues histidine, cysteine, methionine, and selenocysteine. Altogether, the obtained results contribute to shed light into the enhanced antitumoral activity of AF-I compared with AF.

## Introduction

**Figure 1:**
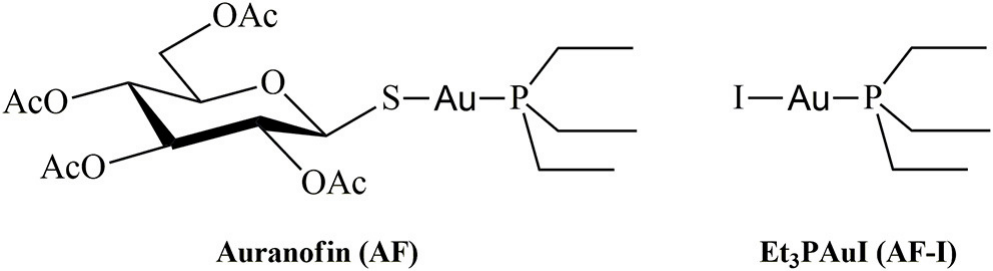
Chemical structures of Auranofin (AF) and Iodo(triethylphosphine)gold(I) (AF-I).

The approval of cisplatin by the FDA in the seventies triggered a huge challenge in the search for innovative metal complexes with improved efficacy and fewer side effects. Within these efforts, several transition metals, including Ag, Cu, Au, and Ru, have been exploited for the synthesis of experimental antitumor compounds (Garbutcheon-Singh et al., 2011; Tamasi et al., 2014; Massai et al., 2016; Pratesi et al., 2016; Ndagi et al., 2017). Among them, gold compounds have attracted growing attention. The leading compound of this family of metallodrugs is auranofin (2,3,4,6-tetra-o-acetyl-L-thio-β-D-glyco-pyranosato-S-(triethyl-phosphine)-gold(I), Figure 1). It is clinically approved for the treatment of rheumatoid arthritis; however, in the frame of the so called “drug repositioning” strategy, in recent years, an extensive reappraisal of its medicinal properties was started unveiling its potential application as antibacterial (Cassetta et al., 2014; Thangamani et al., 2016; Marzo et al., 2018), antiviral (AbdelKhalek et al., 2019; Marzo and Messori, 2020), antifungal, and antiparasitic agent (Bulman et al., 2015; Wiederhold et al., 2017). Moreover, the remarkable *in vitro* antiproliferative properties of this compound fueled the widespread studies of its antitumor activity. AF was found to be efficient in inducing cells apoptosis in various types of human cancers: ovarian (Marzano et al., 2007; Guidi et al., 2012), prostate (Shin et al., 2017), breast (Kim et al., 2013), lung (Liu et al., 2013; Fan et al., 2014; Li et al., 2016; Hu J. et al., 2018), bone (Topkas et al., 2016), and blood (Talbot et al., 2008; Nakaya et al., 2011; Habermann et al., 2017). It has also been included in a few clinical trials as an experimental antineoplastic agent (www.clicalTrials.gov). Unlike the platinum antitumor complexes, which target the nuclear DNA to exert their anticancer action, AF, as the majority of gold complexes, has a greater affinity for sulfur and selenium-containing proteins. For instance, AF binds extensively and quickly to albumin. Accordingly, beyond albumin (Roberts et al., 1996; Pratesi et al., 2018), several non-genomic targets able to coordinate gold metal fragments have been hypothesized to be involved in determining its anticancer activity. Among them are the proteasome system (Micale et al., 2014), the NF-κB protein complex (Nakaya et al., 2011; Hu M. et al., 2018), and thioredoxin reductase (TrxR) (Marzano et al., 2007; Pratesi et al., 2010, 2014; Harbut et al., 2015). The latter is believed to be the main target. Indeed, the inhibition of TrxR by AF through coordination of the gold fragment at the level of the redox-active site, may lead to oxidative stress in cells, eventually leading to apoptosis (Marzano et al., 2007; Harbut et al., 2015). Nevertheless, AF's mechanism of action is not yet fully understood at the molecular level despite multiple experimental and computational studies (Zou et al., 2000; Howell, 2009; Di Sarra et al., 2013; Marzo et al., 2017, 2019). There is a wide consensus that the true pharmacophore is the [Au(PEt_3_)]^+^ cation, whereas the thiosugar ligand is believed to act predominantly as a carrier ligand (Marzo et al., 2017, 2018). [Au(PEt_3_)]^+^ is generated after release of the thiosugar ligand and it is capable to bind the key cellular targets triggering the pharmacological effect (Zou et al., 2000; Marzo et al., 2017, 2019; Zoppi et al., 2020).

In recent years, some of us have started to explore the mechanistic implications determined by the replacement of the thiosugar moiety with different ligands, mainly halides or pseudohalides. The inclusion of halides in the place of the thiosugar ligand could modify the lipophilicity of this metallodrug and thus amplify its bioavailability. Nevertheless, the biologically active [Au(PEt_3_)]^+^ moiety is not affected by this modification. A systematic and comparative investigation concerning the effects on the anticancer properties of the structural modifications was also performed (Cirri et al., 2017, 2019; Marzo et al., 2017, 2019). Through this approach, the complex Au(PEt_3_)I, i.e., the analog of AF where the thiosugar moiety is replaced by iodide, was eventually selected thanks to the very favorable biochemical profile accompanied by a remarkable anticancer activity (Figure 1). Indeed, this analog has a potent cytotoxic effect *in vitro* in colorectal and ovarian cancer, and, more importantly, it induces a rapid tumor mass reduction leading to an almost complete remission in an orthotopic mouse model of ovarian cancer combined with no side effects (Marzo et al., 2017, 2019).

These premises spurred us to further explore the mechanisms of interaction with relevant biological targets of AF-I in comparison with AF. We decided to use a multi-technique experimental approach (NMR, IR and ESI-MS) paired with theoretical calculations (DFT) to study the reactivity of these two gold compounds toward relevant amino acids (His, Met, Cys, and Sec) or their molecular models. This strategy is convenient because it allows us to formulate a quite comprehensive picture for the reactivity and for the mechanisms of activation of metallodrugs in the presence of biological targets or their simplified models avoiding the limits of using a single technique or approach. Also, the multi-technique strategy turned out to be very useful since the studied systems did not responded in the same manner to all the mentioned analytical techniques. In fact, in some cases not all of these were able to highlight the adducts formation, requiring a further interrogation grounded on another instrumental method. His, Met, Cys, and Sec aminoacidic residues were selected since they are generally recognized as the likely binding site for the coordination of gold complexes to proteins in reason to their nature of soft ligands (Abhishek et al., 2019; Pratesi et al., 2019). Results reveal differences in the reaction profiles between AF and AF-I in particular toward both cysteine and selenocysteine while -in our experimental conditions- no reactivity occurs toward His and Met. Furthermore, insights into the kinetic and thermodynamic features for the explored reactivities was gained. Altogether, the information achieved may turn crucial in shedding light into the relevant mechanisms of reaction for the enhanced antitumor activity of AF-I compared to AF.

## Results

### NMR Spectroscopy

We first explored comparatively the reactivity of AF-I and AF through NMR experiments. Owing to solubility issues for both the investigated complexes, the NMR experiments were carried out in the presence of 50% of methanol-d_4_ and 50% of 500 mM sodium bicarbonate buffer (pH=7.8). It is worth reminding that AF and AF-I possess a high stability in a wide array of organic solvents including MeOH (Marzo et al., 2017, 2018), and no side reactions involving the solvent are thus expected. Following addition of AF or AF-I, ^31^PNMR spectra were recorded immediately at sample preparation (*t* = 0), and after 24 h (*t* = 24 h). Table 1 summarizes the results obtained for either AF or AF-I.

**Table 1:** Results obtained by ^31^PNMR experiments for the incubation at different time intervals (*t* = 0 and *t* = 24 h) of AF and AF-I with His, Met, Cys, or Sec.

	**His**		**Met**		**Cys**		**Sec**	
	**t_**0h**_**	**t_**24h**_**	**t_**0h**_**	**t_**24h**_**	**t_**0h**_**	**t_**24h**_**	**t_**0h**_**	**t_**24h**_**
AF	^-^	^-^	^-^	^-^	^#^	^#^	Au(PEt_3_)-S-DTT (39.1 ppm)^*^ Au(PEt_3_)-Se-CH_2_-R (62.6 ppm)^**^	Au(PEt_3_)-Se-CH_2_-R (62.3 ppm)^**^
AF-I	^-^	^-^	^-^	^-^	Au(PEt_3_)-S-CH_2_-R (38.7 ppm)^***^	Au(PEt_3_)-S-CH_2_-R (38.7 ppm)^***^	Au(PEt_3_)-S-DTT (39.1 ppm)^*^ Au(PEt_3_)-Se-CH_2_-R (62.7 ppm)^**^	Au(PEt_3_)-Se-CH_2_-R (62.9 ppm)^**^

- *No adduct formation*.

* *Adduct with dithiothreitol (DTT)*;

** *Adduct with Se of selenocysteine*;

*** *Adduct with S of cysteine (Cirri et al., 2019)*.

# *^31^P signal of Auranofin overlaps that of Au(PEt_3_)-S-CH_2_-R, making impossible the study of the reactivity*.

*R= –CH(NH_2_)COOH*.

For the incubation in the presence of His or Met both complexes appear unreactive—or only a very small amounts of them reacts producing a quantity of the adducts that is not detectable through NMR analysis. Indeed, for AF, the ^31^PNMR signal at 39 ppm, attributable to the phosphorus in the neutral complex (Marzo et al., 2017) remains stable even for incubation up to 24 h. Similarly, the ^31^PNMR signal of the phosphorus of the iodide analog, falling at ~41 ppm, remains unaltered. Only in the case of the incubation in the presence of Met, AF-I undergoes a partial scrambling reaction producing the signal at ~47.5 ppm assignable to the cationic species [Au(PEt_3_)_2_]^+^, according to the already reported mechanism (Marzo et al., 2017). Conversely, the comparative analysis unveils substantial differences in the reactivity profiles of the two drugs when incubated with Cys and Sec. In this case AF-I readily reacts with the thiol moiety of cysteine, producing the adduct where the iodide ligand is displaced, and the ^31^PNMR signal of the adduct appears at ~39 ppm. Moreover, the fast adduct formation in the case of AF-I, is further supported by the production of bubbles upon addition of the complex to the amino acid solution in the NMR tube. In fact, CO_2_ bubbling takes place as neutralization of hydriodic acid by the bicarbonate buffer. On the other hand, it was not possible to elucidate the reactivity of AF against Cys by ^31^PNMR because of the overlap of the signal of the hypothesized adduct Au(PEt_3_)-S-CH_2_-R (where R= –CH(NH_2_)COOH) with that of AF. When AF and AF-I are incubated with Sec, a very similar reactivity occurs. At *t* = 0 h, in both cases two signals are detectable at ~39 and 62.7 ppm. The former is attributable to the adduct of the metal cationic fragment [Au(PEt_3_)]^+^ with DTT necessary to reduce the Se-Se bridge of the selenocystine (Pratesi et al., 2010, 2014; Fabbrini et al., 2019). The latter signal is attributable to the adduct of the same cationic metal fragment with Sec (Ashraf and Isab, 2004). After 24 h of incubation, only the signal at 62.7 ppm appears in the ^31^PNMR spectra in the case of AF as well as for AF-I, indicating that the transient species where DTT coordinates the gold-containing fragment, is quantitatively converted to the gold-selenium adduct (see SI for the NMR spectra). Even in the case of incubation of AF and its analog with Sec, the production of CO_2_ -when the two reactants are mixed together- confirms a rapid interaction with consequent release of hydroiodic acid.

### FT-IR Spectroscopy

**Figure 2:**
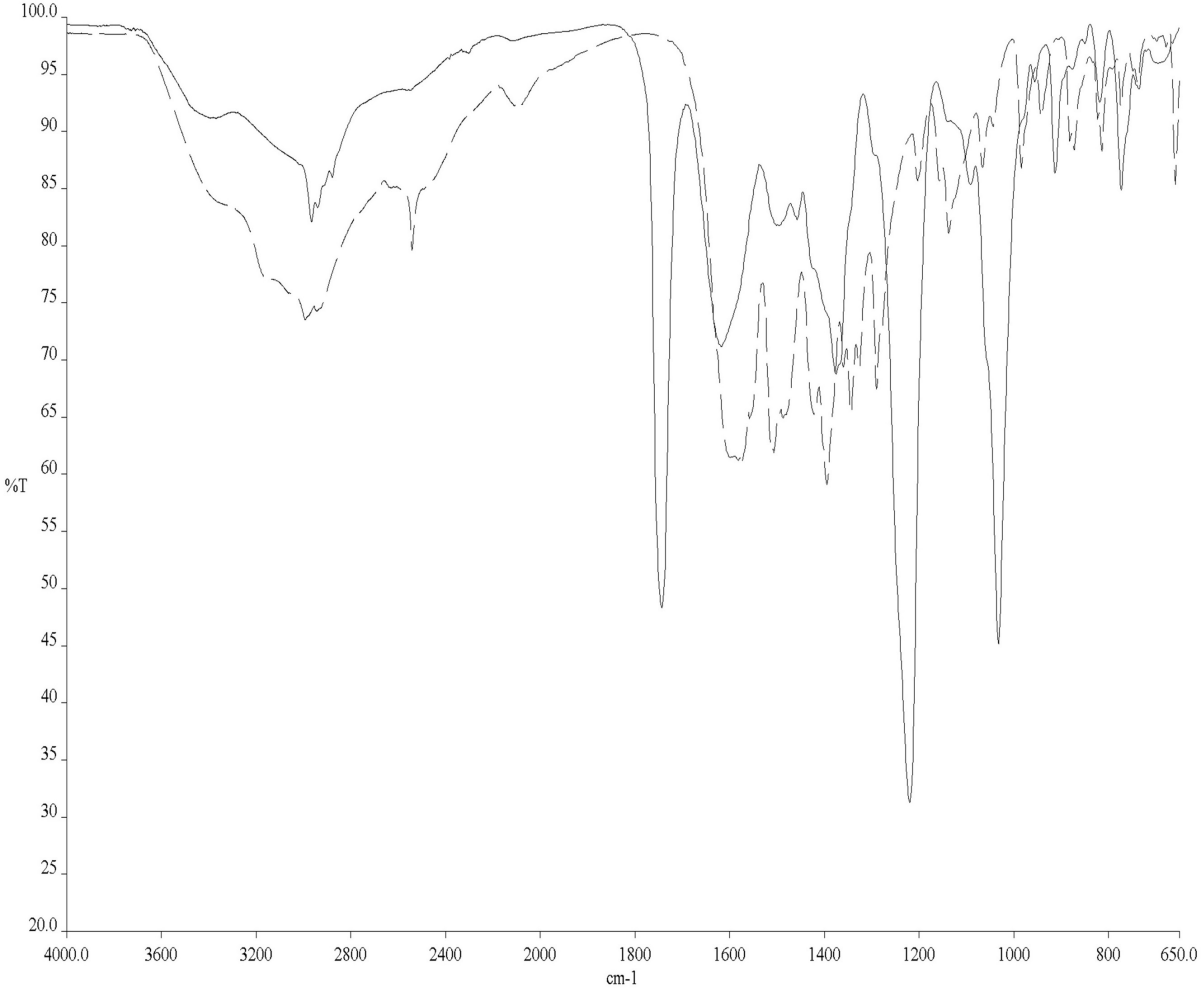
ATR-FTIR spectra of Cys (dashed line) and Cys/AF adducts (solid line).

To avoid the problems arising from ^31^PNMR chemical shift overlap in the NMR experiments, the reactivity of Cys with AF and AF-I was further investigated through solid state ATR-FTIR spectroscopy. More precisely, Cys was incubated for 24 h in the presence of AF and AF-I in the same experimental conditions used for the ^31^PNMR spectroscopy and then the two collected spectra were compared with the IR profile of untreated Cys. In both cases, the infrared spectra showed the disappearance of the absorption band located at 2,542 cm^−1^, assigned to Cys -S-H stretching mode (Zhang et al., 2020). In Figure 2 is reported the superimposition between the IR spectra of the unreacted Cys and of the Cys-AF adduct, and the disappearance of the diagnostic band is evident. These results are in agreement with those obtained with ^31^PNMR in the case of AF-I and confirm a similar reaction pattern of AF toward the Cys -SH moiety.

### ESI Mass Spectrometry

**Figure 3:**
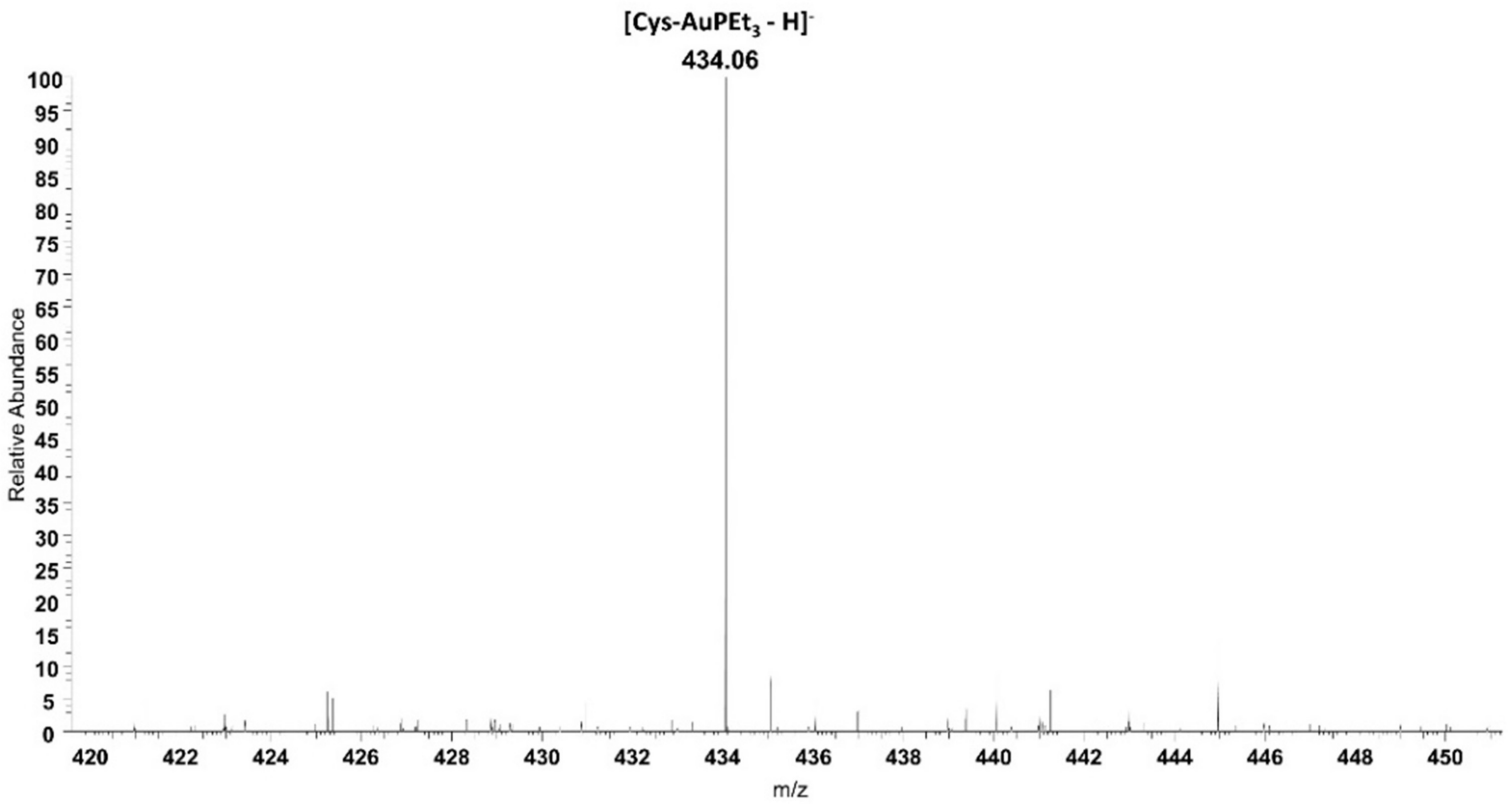
ESI mass spectrum of auranofin (10^−5^ M) incubated for 24 h at 37 °C with cysteine (1:1 metal to amino acid ratio), in ammonium acetate buffer 500 mM pH=6.8 in presence of 50% MeOH.

Beyond IR spectroscopy, additional evidence for the reactivity of AF with cysteine was obtained through ESI-MS experiments. To this aim, auranofin was incubated in the presence of cysteine. After 24 h of incubation a peak at 434.06 *m/z* assignable to the adduct where the cationic fragment [AuPEt_3_]^+^ is coordinated to the amino acid appeared (Figure 3). This evidence strengthens and supports the results already obtained with IR experiments. Similarly, also the reactivity of selenocysteine was assessed with both AF and AF-I (see Supplementary Information). Since this amino acid is commercially available as selenocystine, an initial reduction step with DTT is required to restore the reactive selenol group. Also in this case, the selenol group reacted with the [AuPEt_3_]^+^ cation from AF and from its iodido derivative. In both cases the signal at 530.02 *m/z* is diagnostic for the Sec reactivity and assignable to the adduct with the [AuPEt_3_]^+^ moiety plus one molecule of MeOH and one ${\text{NH}}_{4}^{+}$ deriving from the reaction buffer. Interestingly, those signals clusters show the characteristic selenium isotopic pattern.

Next, we investigated more in depth the reactivity of both AF and AF-I with His and Met. In fact, the lack of adducts formation could be the consequence of a slower kinetics in the reaction of the two gold complexes with these amino acids compared with Cys and Sec. This may lead to a relatively scarce adducts formation in 24 h that might be non-detectable with NMR analysis. In this case, also the IR analysis failed due to the lack of characteristic bands that can be followed to determine His and Met reactivity. Thus, we performed ESI-MS experiments also for AF and AF-I incubated with the two latter amino acids. Results confirmed the lack of reactivity for these two target molecules with AF and AF-I, further supporting the findings already published by some of us regarding the reactivity of auranofin and its derivatives toward protein targets (Landini et al., 2017; Pratesi et al., 2018; Zoppi et al., 2020).

Both in case of His and Met, the spectra recorded with AF only show a main peak at 679.13 m/z corresponding to the unreacted auranofin. At variance, in the case of AF-I the peaks at 433.15, 442.96, and 459.99 *m/z* belong to [Au(PEt_3_)_2_]^+^, AF-I, and [AF-I+NH_4_]^+^, respectively. The [Au(PEt_3_)_2_]^+^ cation is a well-known species deriving from the ligand scrambling reaction of AF-I (Marzo et al., 2017). All these spectra are reported in the Supplementary Material.

### DFT Calculations

**Scheme 1:**
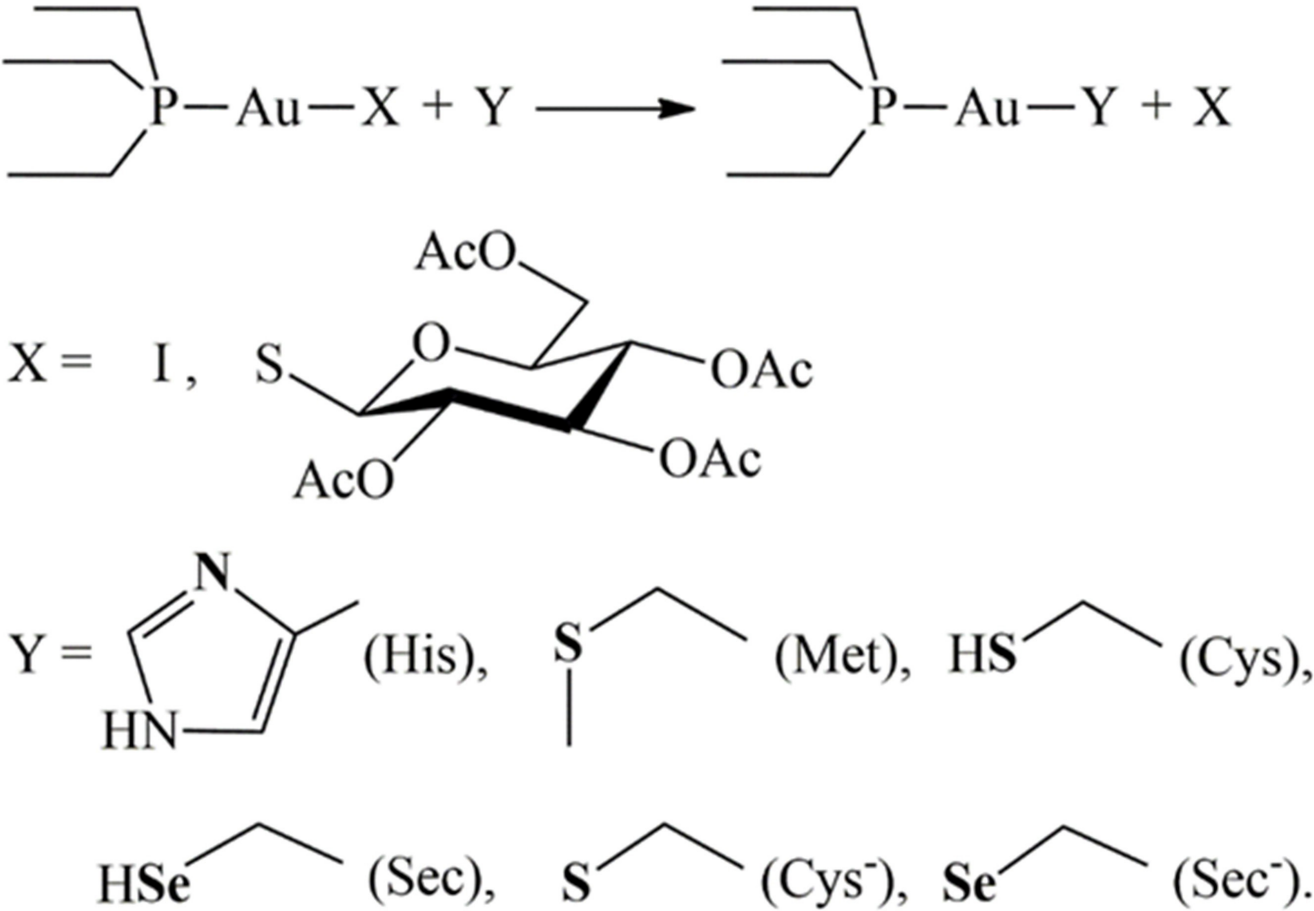
Reaction scheme and considered nucleophiles. Attacking atoms in bold.

Next, we have computed the reactivity of the neutral AF and AF-I complexes with water and with the side chains of histidine, methionine, cysteine, and selenocysteine residues. We have used simplified models of these residues (Scheme 1). Each side chain was represented by the nucleophilic group, while the rest of the chain was represented by the ethyl group.

**Figure 4:**
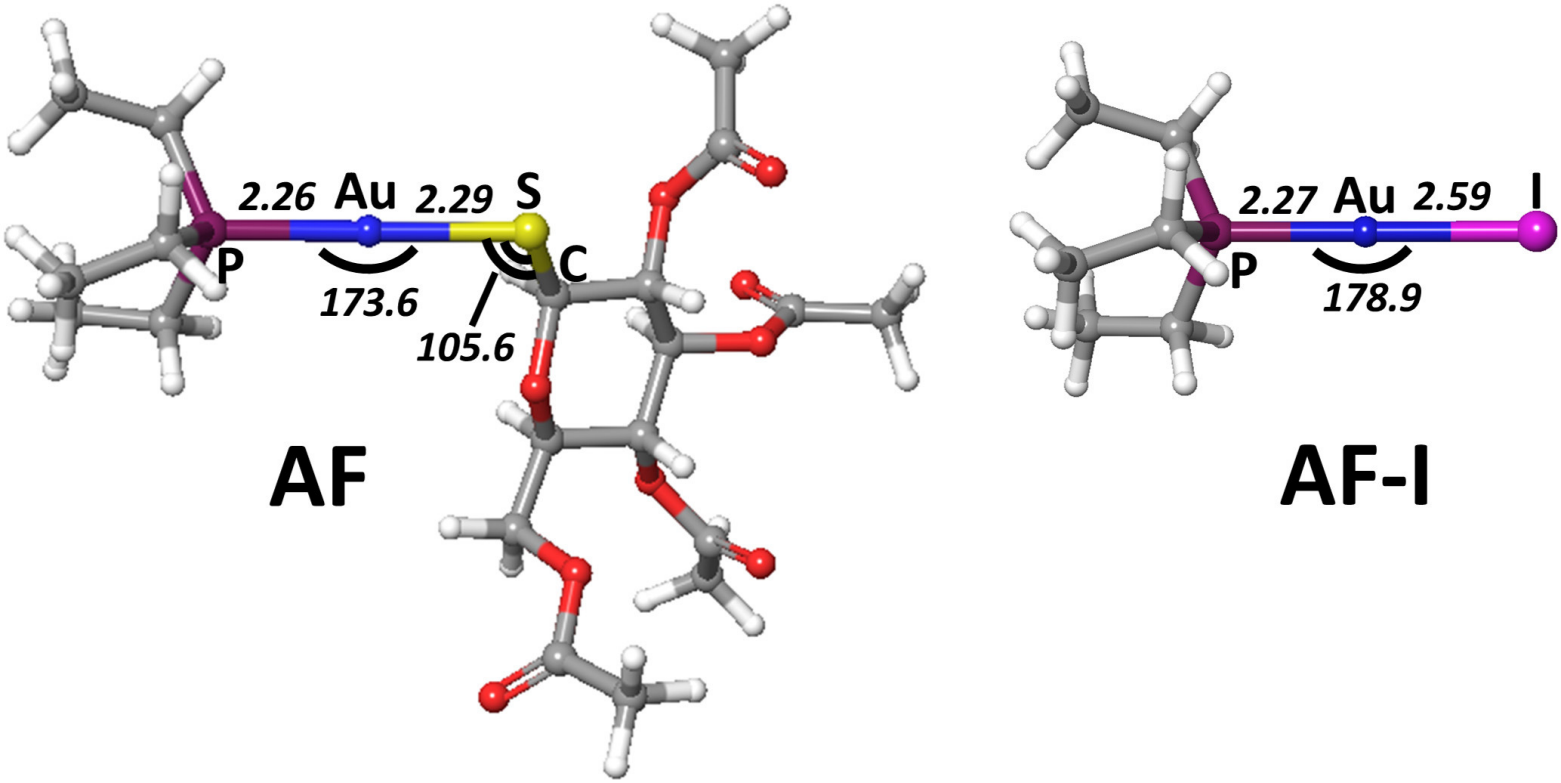
Representation of the experimentally characterized structures of AF and AF-I complexes, the respective crystallographic cartesian coordinates are extrapolated from (Hill and Sutton, 1980; Marzo et al., 2017). All distances are in angstroms, all angles in degrees.

In order to gain deeper insight into the binding mode of the AF and AF-I complexes with protein targets and their binding preference, we have taken advantage of a computational strategy aimed at evaluating the thermodynamic and kinetic basis of the proposed mechanistic hypotheses. A preliminary geometry optimization of the AF and AF-I complexes with various density functionals yield structures in good agreement with the crystallographic data (Hill and Sutton, 1980; Marzo et al., 2017). Indeed, the Au-P, Au-S, and Au-I bond distances fall within an error of 0.08 Å, and P-Au-S and P-Au-I angles are within 7°: in particular, the errors for all the calculated geometrical parameters indicate that the CAM-B3LYP functional provides the best description for these molecular structures (see Table 2, Figure 4) and will be employed in all subsequent calculations.

**Table 2:** Assessment of five density functionals for the geometry optimization via comparison of experimental and calculated values for structural parameters of AF and AF-I complexes (Figure 1).

**Density functionals**	**AF**				**AF-I**			Errors^*^
	**Distances**		**Angles**		**Distances**		**Angles**	
	**Au-P**	**Au-S**	**P-Au-S**	**Au-S-C**	**Au-P**	**Au-I**	**P-Au-I**	
wB97X	2.30	2.34	178.8	103.1	2.30	2.62	179.4	8.35
M06L	2.30	2.37	178.5	98.3	2.30	2.64	180.0	9.20
B3LYP	2.32	2.36	178.5	103.7	2.32	2.65	179.8	8.90
CAM-B3LYP	2.30	2.34	178.3	103.7	2.30	2.62	179.6	8.05
B3LYP-D3	2.30	2.37	177.8	99.1	2.31	2.65	179.9	8.10
Exp [39, 71]	2.26	2.29	173.6	105.6	2.27	2.59	178.9	-

*All distances are in angstroms and all angles in degrees. No reactant-adducts for the reactions of AF/AF-I with deprotonated cysteine and selenocysteine could be optimized*.

DFT calculations were therefore carried out on the thermodynamics and the kinetics of the ligand exchange reactions between the thiosugar of AF or the iodide in case of AF-I and the selected nucleophilic molecules. Moreover, simplified models of the amino acid residues side chains (Scheme 1) were conveniently used for the theoretical study as previously reported (Tolbatov et al., 2020b). The amino acid capped forms, although chemically resembling the protein residues, are connected to the side chain nucleophilic group through hydrocarbon chains of different lengths, thus leading to a large variation in the ligand's size reacting with metal complex. This latter aspect may bias the solvation free energies calculation, thus affecting the comparison of amino acid reactivity. All residues were assumed to be in their most stable protonation state at pH=7.8, as expected on the pKa of the side chain groups, i.e. neutral for histidine (pKa=5.9) and methionine and anionic for selenocysteine (pKa=5.2), for which we also considered the neutral form for sake of completeness). For cysteine (pKa=8.3) we considered both the neutral form, expected to be the most stable at pH 7.8, and the anionic form, which is still present in low concentrations (ca. 30%). Preliminary, we considered the hydrolysis reaction with substitution of the thiosugar or iodido ligand by a water molecule to probe the stability of AF and AF-I complexes in biological fluids. The calculated reaction free energy values for the aquation reaction of AF and AF-I are 18.3 and 23.0 kcal/mol, respectively. These values indicate that the reaction is negligible at physiological temperature, and that both complexes are likely to attack the biological targets in their administered form. This result is consistent with the experimental evidences showing as the solutions of both AF and AF-I complexes are stable at room temperature even for very long incubation times (days) (Marzo et al., 2017, 2018).

The ligand exchange reactions on AF and AF-I were investigated by assuming an associative interchange mechanism where both reagents and products form stable non-covalent adducts prior/after the reaction takes place. We have thus optimized the geometries of the reactant adducts (RA), transition states (TS), and product adduct (PA) intermediates and their energies with respect to the isolated species have been evaluated (see below). Calculations indicated a very low stability of RA and PA adducts, which cannot even be optimized for cysteine or selenocysteine side chains (*vide infra*). The activation enthalpies and free energies have been calculated as the difference between TS and the lowest between reactants and reactant adducts, while the reaction enthalpies and free energies have been calculated as the difference between reagents and products infinitely apart.

**Figure 5:**
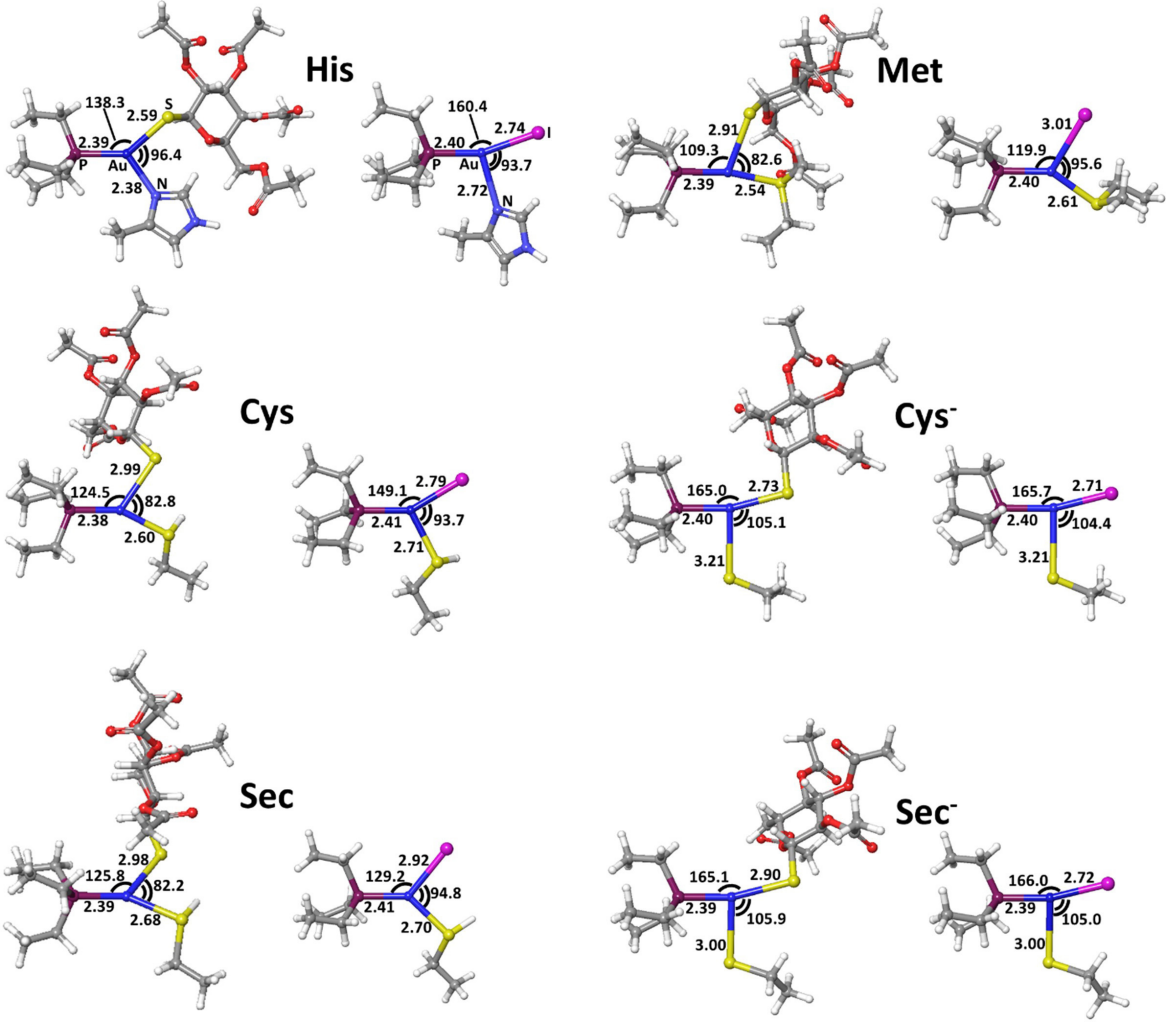
Transition states for thiosugar or iodide substitution by all considered amino acids.

The calculated values of reaction enthalpy and free energy for the examined ligand exchange processes (Table 3 and Figure 5) allow us to establish the thermodynamic preferences for the Au(I) binding toward the considered protein residues side chains. Indeed, we found that the substitution of iodide is always much more favored than the one of thiosugar, the latter being featured in higher reaction enthalpy and free energy values. Calculations also indicated that the reactions of both the Au(I) complexes with the considered neutral amino acid side chain are endothermic and endergonic, 9–10 kcal mol^−1^ for His and 16–17 kcal mol^−1^ for Met, Cys, and Sec, whereas the reactions with deprotonated cysteine and selenocysteine side chains are slightly exothermic and exergonic, by 3–4 kcal mol^−1^ for Cys^−^ and 2–3 kcal mol^−1^ for Sec^−^. Based on the calculated thermodynamics, we can eventually state that the reaction trend for both AF and AF-I with the examined side chain models can be rationalized as Cys^−^ > Sec^−^ >> His > Met ≈> Sec ≈ Cys, thus remarking the importance of the protonation state of the nucleophile. These results clearly show that the thiosugar or iodide substitution reaction is thermodynamically unfavorable for neutral amino acids, His, Met, Cys, and Sec and is therefore not expected to occur, while it is possible only for Cys and Sec in their anionic forms.

**Table 3:** Enthalpies and Gibbs free energies for reaction of AF/AF-I with various biomolecular targets in solution.

**Biomolecular target/Side chain of protein residue**	**Metallodrug**	**R->P**		**R->TS**		**RA->TS**	
		**ΔH**	**ΔG**	**ΔH**	**ΔG**	**ΔH**	**ΔG**
His	AF	9.4	9.6	11.3	25.6	10.0	14.4
	AF-I	5.7	9.8	5.1	16.3	3.3	7.4
Met	AF	14.8	16.4	18.7	32.4	18.8	23.6
	AF-I	11.2	16.6	5.9	17.8	5.2	10.1
Cys	AF	18.1	17.4	17.2	26.7	16.2	18.7
	AF-I	14.4	17.6	5.9	15.5	5.7	7.7
Sec	AF	15.5	16.8	17.9	27.2	16.6	19.2
	AF-I	11.9	17.0	5.9	16.3	6.0	9.0
Cys^−^	AF	−2.4	−3.9	11.7	21.3	–^*^	–
	AF-I	−6.0	−3.7	2.8	12.0	–	–
Sec^−^	AF	−0.9	−2.5	3.4	21.2	–	–
	AF-I	−4.4	−2.2	2.1	14.1	–	–

* *No reactant-adducts for the reactions of AF/AF-I with deprotonated cysteine and selenocysteine could be optimized*.

The reactivity of AF and AF-I toward molecular models for the side chain of His, Met, Cys, and Sec was then addressed by calculating the corresponding values of activation enthalpy and free energy.

The calculated transition state structures for the reaction of either AF or AF-I with the neutral amino acids, His, Met, Cys, and Sec, show a roughly planar trigonal coordination of the Au(I) center with entering group-metal-leaving group angles in the 82-96° range, whereas the transition states for the anionic Cys^−^ and Sec^−^ models species, show earlier transition state structures with almost T-shaped and with entering group-metal-leaving group angles of 104-105° (Figure 5).

The calculated activation free energies in Table 3 show for neutral amino acids barriers much higher for AF than for AF-I, ca. 10–15 kcal mol^−1^. Although the barriers for the iodide substitution in AF-I, 15–18 kcal mol^−1^, are low (too low to foresee relatively fast substitution reactions), these are not expected to occur due to the unfavorable thermodynamics as discussed above. For thiosugar substitution by neutral amino acids, the activation barriers are relatively high, 25–32 kcal mol^−1^, making these reactions not only thermodynamically but also kinetically unfavorable. On the other hand, significantly lower activation barriers were calculated for the reaction of either AF or AF-I with cysteine and selenocysteine in their anionic form. Namely, the calculated activation free energy values for substitution of iodide ligand by Cys^−^ and Sec^−^ were found to be particularly low 12.0 and 14.1 kcal mol^−1^, respectively, while those for thiosugar substitution were found 21.2 and 21.3 kcal mol^−1^, respectively, low enough to guarantee reaction half times of only a few minutes.

**Figure 6:**
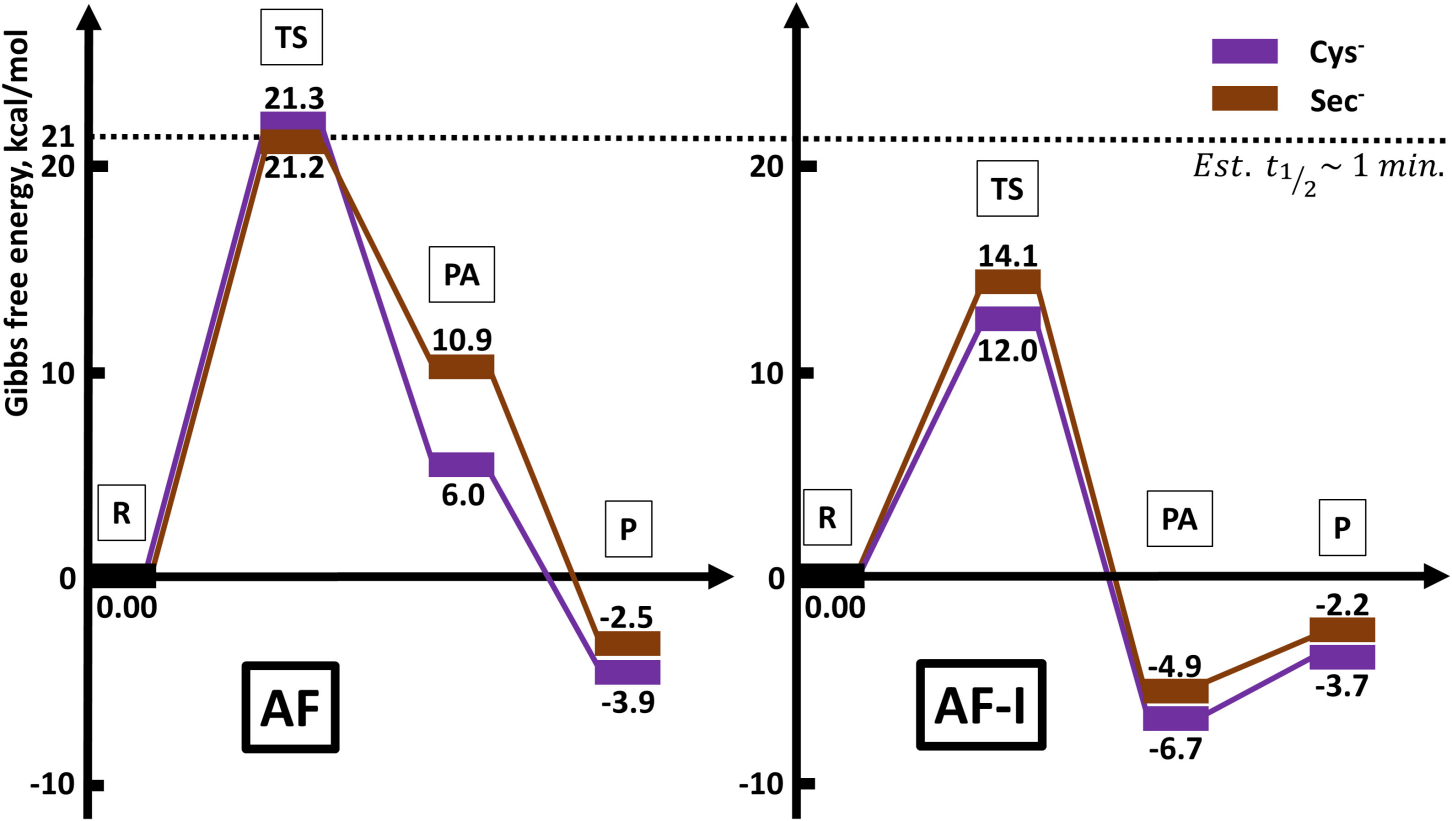
Reaction profiles for AF and AF-I interacting with neutral His and Met and anionic Cys^−^ and Sec^−^ nucleophiles.

An overall picture of the thermodynamics and of the ligand exchange reactions for the anionic Cys^−^ and Sec^−^ amino acids, is provided in Figure 6 and clearly shows as only for the anionic species thiosugar or iodide substitution is both thermodynamically and kinetically feasible, in agreement with experimental evidence.

## Discussion and Conclusions

Starting from our previous findings on the reactivity of AF and its analogs toward protein targets (Pratesi et al., 2014, 2018; Marzo et al., 2017, 2019; Cirri et al., 2019; Zoppi et al., 2020), here we have extended the investigation—at the molecular level—on the mode of action of these medicinally relevant gold(I) compounds. Specifically, we have focused our attention on AF and its analog where the thiosugar moiety is replaced by the iodide ligand. All the previous data seem to converge toward an unambiguous predilection of these gold (I) complexes for the free and solvent accessible cysteine and selenocysteine amino acid residues of the studied proteins. Nonetheless, some other residues i.e., methionine and histidine may represent, at least theoretically, a possible binding site for these compounds.

Accordingly, in this work, we have further elucidated the reactivity of AF and its promising anticancer analog AF-I through a multi-techniques approach involving NMR, FTIR and ESI-MS.

Since the [AuPEt_3_]^+^ cation has been recognized as the reactive fragment, the ^31^PNMR can be considered the election technique to characterize adducts formation with the selected amino acids. Hence, an extensive experimental work has been carried out demonstrating that AF and AF-I react quite completely with Sec after 24 h. Also, in case of Cys, the same reactivity has been highlighted with AF-I. Unfortunately, the reaction between Cys and AF did not produce any appreciable shift of the ^31^P signal and makes difficult to assess adduct formation.

In this case, the reaction has been followed with the ATR-FTIR since Cys possesses a diagnostic band at 2,542 cm^−1^ due to the Cys -S-H stretching. The disappearance of this band is evidential for the amino acid reactivity because the formed -S-Au bond stretching falls below 200 cm^−1^. Moreover, this band result disappeared entirely, leaving us to guess a near quantitative reaction after 24 h.

This behavior has been further assessed with ESI-MS; the obtained spectra show as the main peaks the signals corresponding to the AF and AF-I adducts with Sec and Cys.

Interestingly, the ^31^PNMR spectra do not show any appreciable chemical shift also in the case of Met and His when reacted with AF and AF-I. In these cases, also the ATR-FTIR is not useful because, similarly to Cys, the two amino acids do not possess a diagnostic band. Thus, we eventually exploited ESI-MS analysis that confirmed the lack of reactivity for the gold complexes with Met and His. Indeed, the only signals present in the spectra are those belonging to AF and to a couple of well-known AF-I scrambling products in MeOH solution in the case of the iodide analog (Marzo et al., 2017).

As a completion of this study, the reactivities of AF and AF-I toward the selected amino acids have been evaluated also with computational methods. We considered the free and unprotected amino acids and their reduced molecular models, i.e. imidazole, methylselenol, and methylthiol. This simplification can be done since the gold(I) complexes are unable to give bidentate coordination between the sidechain and the carboxyl or amine group of the amino acid.

Computational studies have often and successfully been applied to describe the reactivity of metals and metallodrugs with proteins (Graziani et al., 2016; Tolbatov et al., 2019b, 2020c). Understanding the binding preference of AF and AF-I would be very useful to fully understand their mechanisms of action *in vivo* and may be useful to conceive novel and more effective anticancer drugs.

On the other hand, computational studies about AF are very few. Density functional theory (DFT) calculations on a very simplified model of AF reacting with methylselenol displayed the preference of breakage of Au-S bond over the Au-phosphine bond that could be concluded from the difference in the gas phase free energy reaction barriers of 11.9 and 22.8 kcal mol^−1^ for the S-CH_3_ and (CH_3_)_3_P exchanges, respectively (Di Sarra et al., 2013). The computational study of the reactivity of AF against S-, Se-, and N-containing amino acids (Dos Santos, 2014) for the substrate and the nucleophiles has supported the conclusion that the replacement of the thiosugar by the protein residues is favored over the replacement of phosphine. This study found that the activation barriers of the amino acids with auranofin follow the trend Lys > His > Sec > Cys according to their activation enthalpy values. In recent studies, reactivity of AF with Cys and Sec was studied via DFT calculations (Howell, 2009; Shoeib et al., 2010) and the overall conclusions were that the Se moiety is the preferred coordination site over the cysteine.

Detailed mechanisms of reaction of AF and AF-I compounds with most amino acids and protein fragments remains largely undisclosed. In this study, we have computed the reactivity of both these complexes with various nucleophiles—side chains of neutral histidine, methionine, and cysteine, and selenocysteine in both neutral and anionic forms.

The high reaction free energy values calculated for the aquation of AF and AF-I indicate that this process is thermodynamically disfavored, and that both complexes are likely to attack their biological targets by the exchange of either thiosugar (AF) or iodide (AF-I).

The theoretical analysis of thermodynamics and kinetics for the ligand exchange processes of AF and AF-I with His, Met, Cys, and Sec side chain models unveiled that thiosugar or iodide substitution is both thermodynamically and kinetically favored only for Cys and Sec, but only in their anionic forms, with barriers for iodide substitution in AF-I significantly lower.

In conclusion, we have presented here a suitable multi-techniques approach allowing to gather mechanistic insights on the reactivity and the mode of action of two promising anticancer agents. The computational analysis is in good agreement with the experimental data, and the energy barrier calculated point out the enhanced reactivity of AF-I with respect to AF toward Cys and Sec. On the other hand, also the lower energetic barriers calculated for the reactivity with Sec and Cys, perfectly support the experimental data obtained, pinpointing a quite exclusive preference for these two aminoacidic residues instead of His and Met. This supports the view that proteins bearing cysteine and selenocysteine residues e.g., thioredoxin reductase, are the likely targets for the pharmacological activity of these gold complexes. In turn, the more pronounced reactivity of AF-I toward Cys and Sec compared to AF might be at the bases of its better anticancer properties *in vivo* (Marzo et al., 2019).

## Materials and Methods

### Materials

Auranofin was purchased from Enzo Life Sciences (Famingdale, New York). AF-I was synthesized as described in (Marzo et al., 2017). Cysteine, histidine, methionine, and selenocysteine were purchased from Merck (Darmstadt, Germany) and used without further purification or manipulation. Water, methanol, and ammonium acetate were of LC-MS grade and were purchased from Merck. MeOD-d_4_ was purchased from Merck with a deuteration grade of 99.9%.

### NMR

AF and AF-I (6.25 mM) were incubated at 37 °C with amino acids or MM (complex to amino acid or MM ratio 1:1) in presence of 400 μL of MeOH and 400 μL of NaHCO_3_ buffer (500 mM) at pH=7.8 and NMR experiments were recorded at increasing time intervals (0 h, 24 h) on a Bruker Ultrashield 400.13 MHz, Bruker Avance III equipped with Probe 5 mm PABBO BB-1H/D Z-GRD Z108618/0049. Calibration was made according to the residual peak of the solvent. Raw data were analyzed using TopSpin 2.1 software (Bruker). In the case of selenocysteine (Sec), this latter was generated *in situ* through the addition of 10 equivalents of DTT (dithiothreitol). Spectra are available in the Supplementary Material.

### FT-IR

Experiments were carried out on Perkin-Elmer SPECTRUM 100 FT-IR Spectrometer equipped with the Perkin-Elmer Universal ATR Sampling Accessory. Samples were prepared as a 30 mM solution of interest compound dissolved in 1:1 MeOH and NaHCO_3_ buffer (500 mM) at pH=7.8 (see Supplementary Material for further details). A drop of solution was deposed on the spectrometer ATR probe and let to completely evaporate in the air before scanning. The acquisition was performed between 4,000 and 650 cm^−1^ with a resolution of 4 cm^−1^.

### ESI-MS

#### Sample Preparation

Stock solutions of AF and AF-I were freshly prepared in LC-MS grade water and methanol (50:50 v/v) to a final concentration of 10^−2^ M. Stock solutions of the amino acids and molecular models were prepared in 500 mM ammonium acetate solution with a pH 6.8 and methanol (50:50 v/v) at 10^−2^ M. As previously described, selenocysteine (Sec) was generated *in situ* through the addition of 10 equivalents of DTT to reduce the Se-Se bond of selenocystine.

For each gold compound/amino acid pair, appropriate aliquots of these stock solutions were mixed and diluted with 500 mM ammonium acetate solution (pH 6.8) to a final amino acid concentration of 10^−4^ M and an amino acid-to-metal molar ratio of 1:1. The solutions were incubated up to 24 h at 37 °C.

#### ESI-MS Analysis

Aliquots were sampled at after 24 h of incubation, diluted to a final concentration of 10^−5^ M with LC-MS grade water and ESI-MS spectra were recorded by direct injection at 5 (for positive polarity) and 7 (for negative polarity) μL min^−1^ flow rate in an Orbitrap high-resolution mass pectrometer (Thermo, San Jose, CA, USA), equipped with HESI source. The working conditions were as follows: positive polarity, spray voltage 3.5 kV, capillary temperature 300 °C, and S-lens RF level 55. The sheath and the auxiliary gases were set at 20 and 3 (arbitrary units), respectively. Negative polarity, spray voltage −3.4 kV, capillary temperature 300 °C, S-lens RF level 55. The sheath and the auxiliary gases were set at 35 and 8 (arbitrary units), respectively. For acquisition and analysis, Xcalibur 4.2 software (Thermo) was used. For spectra acquisition a nominal resolution (at *m/z* 200) of 140,000 was used.

### Computational

All calculations were performed with the Gaussian 09 A.02 (Gaussian 09 | Gaussian.com^1^) quantum chemistry packages. Optimizations, electronic and solvation energy evaluations were carried out in solvated phase (C-PCM) (Barone and Cossi, 1998; Cossi et al., 2003) and by using the density functionals reported on Table 1 (optimization) and Table 2 (electronic and solvation energy) as described below.

All geometrical optimizations have been carried out with the LANL2DZ basis set (Dunning and Hay, 1977; Hay and Wadt, 1985; Roy et al., 2008), whereas single-point electronic and solvation energy calculations were calculated with the LANL08(f) effective core potential for gold (Hay and Wadt, 1985; Ehlers et al., 1993; Roy et al., 2008) and the basis set 6-311++G^**^ for other elements (Krishnan et al., 1980; McLean and Chandler, 1980; Francl et al., 1982; Clark et al., 1983; Spitznagel et al., 1987). We used the DFT functionals in a preliminary geometry benchmarking study B3LYP (Becke, 1993; Stephens et al., 1994), M06L (Zhao and Truhlar, 2006), and range corrected wB97X (Chai-Da and Head-Gordon, 2008), B3LYP-D3 (Grimme et al., 2010), and CAM-B3LYP (Yanai et al., 2004). DFT functionals are known to give a good description of geometries and reaction profiles for transition-metal-containing compounds (Ciancetta et al., 2012; Paciotti et al., 2018, 2019; Todisco et al., 2020) including Au(I) (Tolbatov et al., 2019a, 2020a) based anticancer compounds. The preliminary benchmarking of performance of these density functionals has shown that CAM-B3LYP yields the geometrical structure slightly more accurate than the other functionals, and it was thus chosen as the best for our calculations.

Frequency calculations were performed to verify the correct nature of the stationary points and to estimate zero-point energy (ZPE) and thermal corrections to thermodynamic properties. Intrinsic reaction coordinate (IRC) calculations were employed to locate reagents and products minima connected with the transition states for each considered reaction step.

Single point electronic energy calculations were performed on the solvated-phase geometries. The C-PCM continuum solvent method was used to describe the solvation (Cossi et al., 2003). It has recently been shown to give considerably smaller errors than those for other continuum models for aqueous free energies of solvation for neutrals, cations and anions, and to be particularly effective for the computations of solution properties requiring a high accuracy of solution free energies (Chen et al., 2019). Solvation free energies, taken as the difference between the solution energies and the gas phase energies, were added to the gas phase enthalpies and free energies values to obtain the corresponding values in the aqueous solution.

## Data Availability Statement

All datasets presented in this study are included in the article/Supplementary Material.

## Author Contributions

AP and TM with the support of IT designed, coordinated, and supervised the whole study. IT and DC carried out theoretical calculations and NMR experiments. AM, CC, and NR revised the paper and suggested improvements and corrections for the theoretical section for which they were responsible with IT. AP carried out the MS experiments with the support of TM. CG and LMe revised the experimental part and suggested improvements and corrections together with DL and LMa. AP, IT, and TM wrote the manuscript. All the authors approved the final version of the manuscript.

## Conflict of Interest

The authors declare that the research was conducted in the absence of any commercial or financial relationships that could be construed as a potential conflict of interest.

## Footnotes

^1^Gaussian 09 Citation | Gaussian.com Available online at: https://gaussian.com/g09citation/ (accessed June 18, 2020).
