# Supplementary material for: Mechanistic Insights Into the Anticancer Properties of the Auranofin Analog Au(PEt3)I: A Theoretical and Experimental Study
Source: Front Chem. 2020 Sep 18;8:812. doi: 10.3389/fchem.2020.00812 (PMC7531625; doi:10.3389/fchem.2020.00812)
Supplement: Supplementary file 1 [file Data_Sheet_1.docx]

Supplementary Material

Mechanistic insights into the anticancer properties of the auranofin analogue Au(PEt_3_)I; a theoretical and experimental study

Iogann Tolbatov, Damiano Cirri, Lorella Marchetti, Alessandro Marrone, Cecilia Coletti, Nazzareno Re, Diego La Mendola, Luigi Messori, Tiziano Marzo^*^, Chiara Gabbiani and Alessandro Pratesi^*^

Index

[NMR spectra 2](#_Toc42870752)

[FT-IR spectra 18](#_Toc42870753)

[ESI-MS spectra 21](#_Toc42870754)

# NMR spectra

#
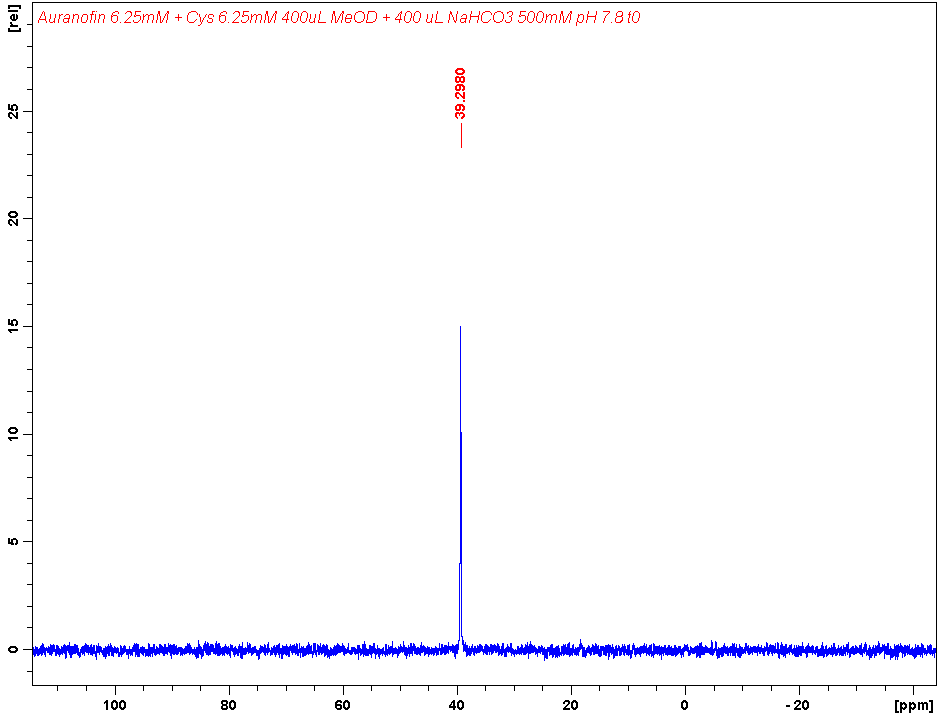


**Supplementary Figure 1.** Auranofin 6.25 mM + Cys 6.25 mM in MeOD-d_4_/Carbonate buffer (500 mM pH 7.8) 1:1. Spectrum recorded at t_0_.


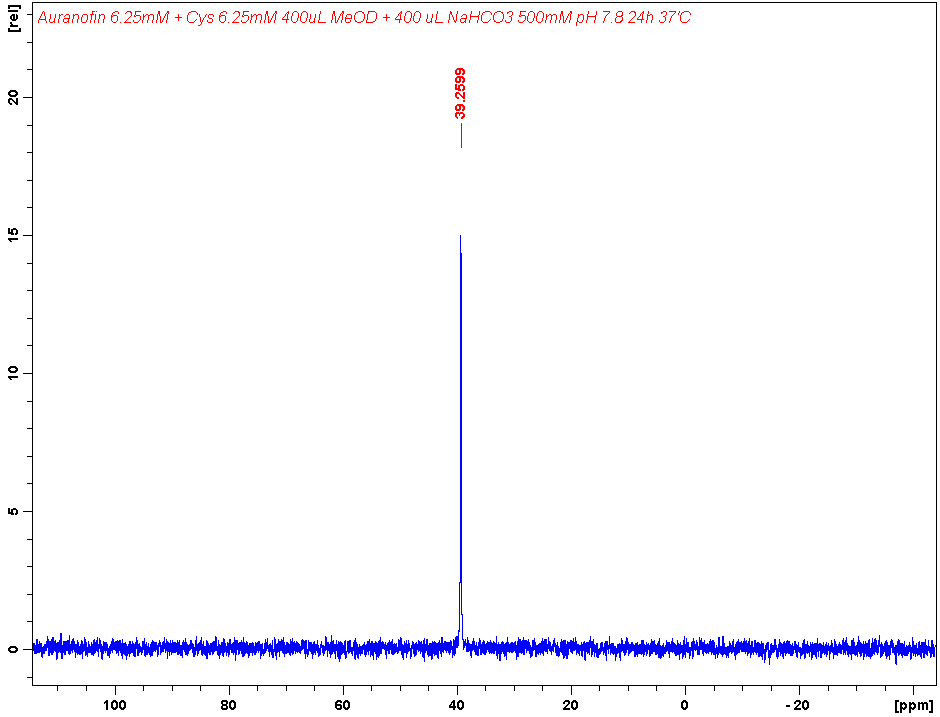


**Supplementary Figure 2.** Auranofin 6.25 mM + Cys 6.25 mM in MeOD-d_4_/Carbonate buffer (500 mM pH 7.8) 1:1. Spectrum recorded after 24h of incubation at 37 °C.


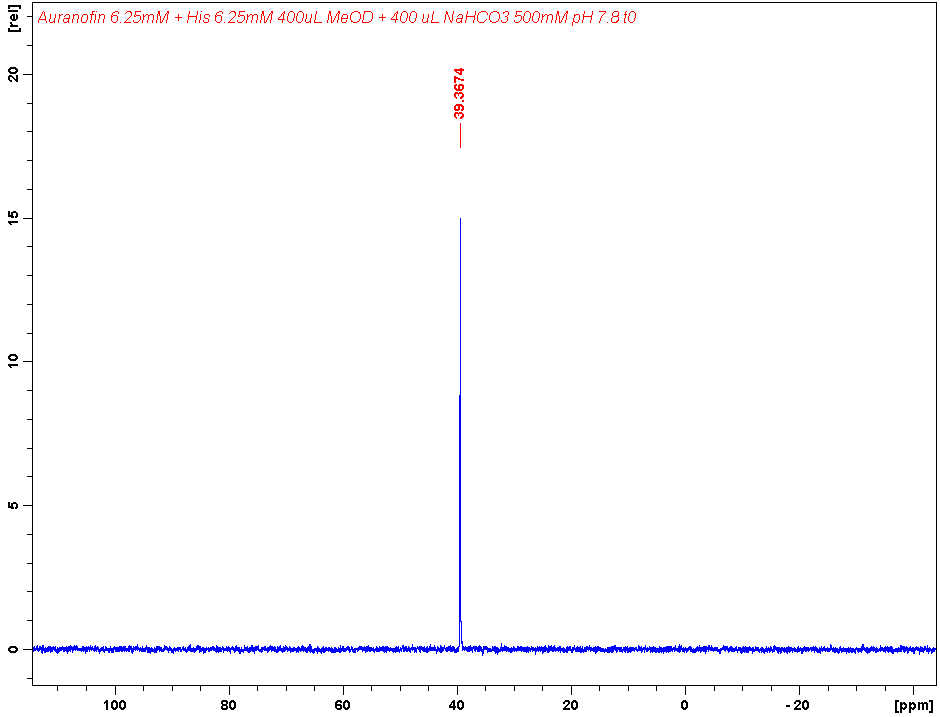


**Supplementary Figure 3.** Auranofin 6.25 mM + His 6.25 mM in MeOD-d_4_/Carbonate buffer (500 mM pH 7.8) 1:1. Spectrum recorded at t_0_.


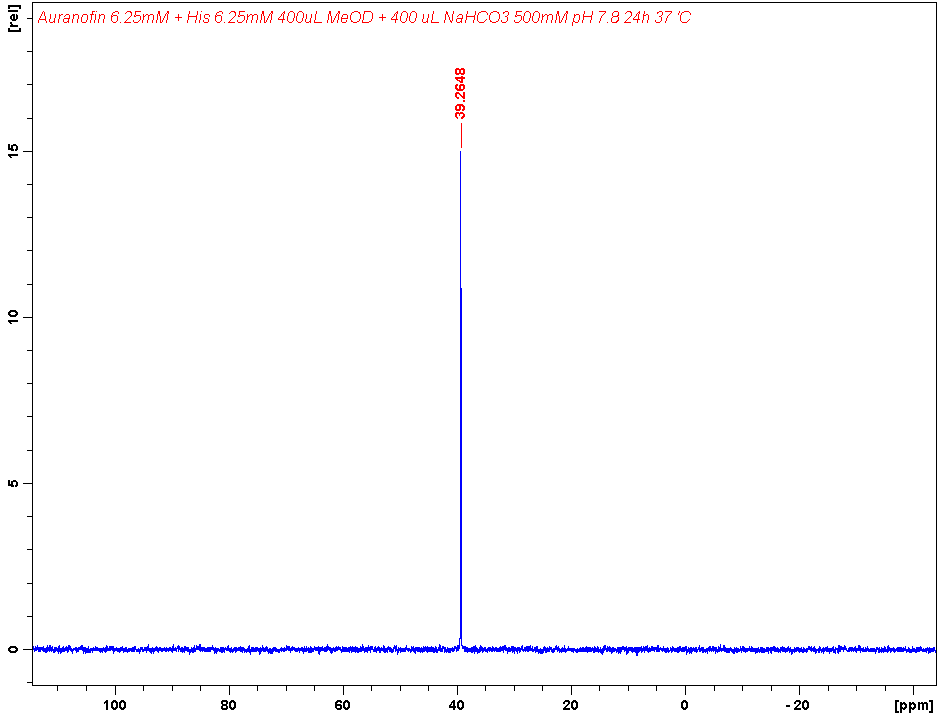


**Supplementary Figure 4.** Auranofin 6.25 mM + Cys 6.25 mM in MeOD-d_4_/Carbonate buffer (500 mM pH 7.8) 1:1. Spectrum recorded after 24h of incubation at 37 °C.


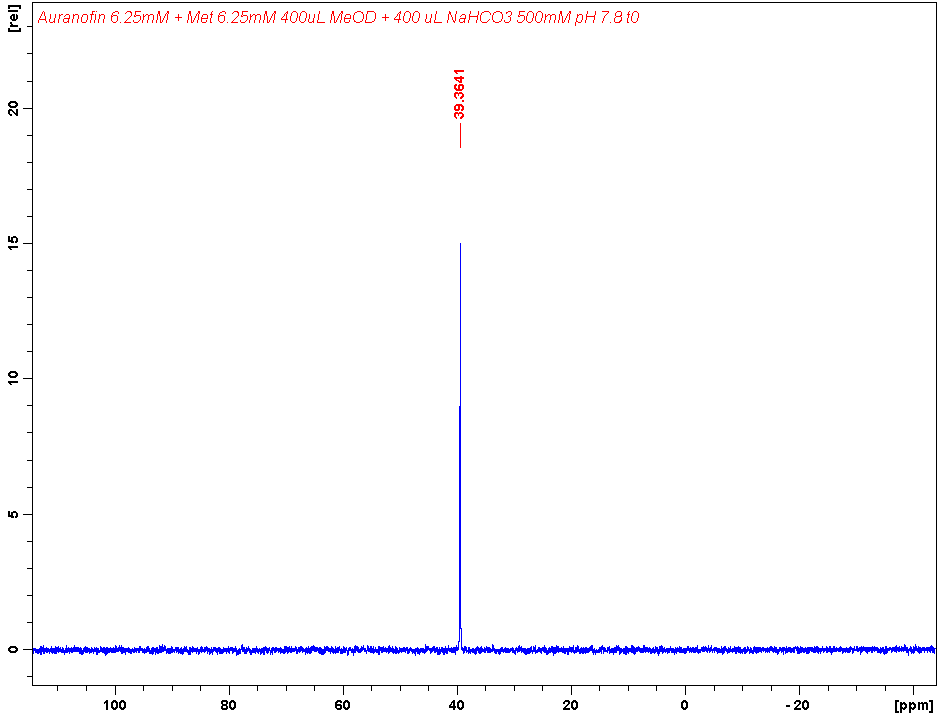


**Supplementary Figure 5.** Auranofin 6.25 mM + Met 6.25 mM in MeOD-d_4_/Carbonate buffer (500 mM pH 7.8) 1:1. Spectrum recorded at t_0_.


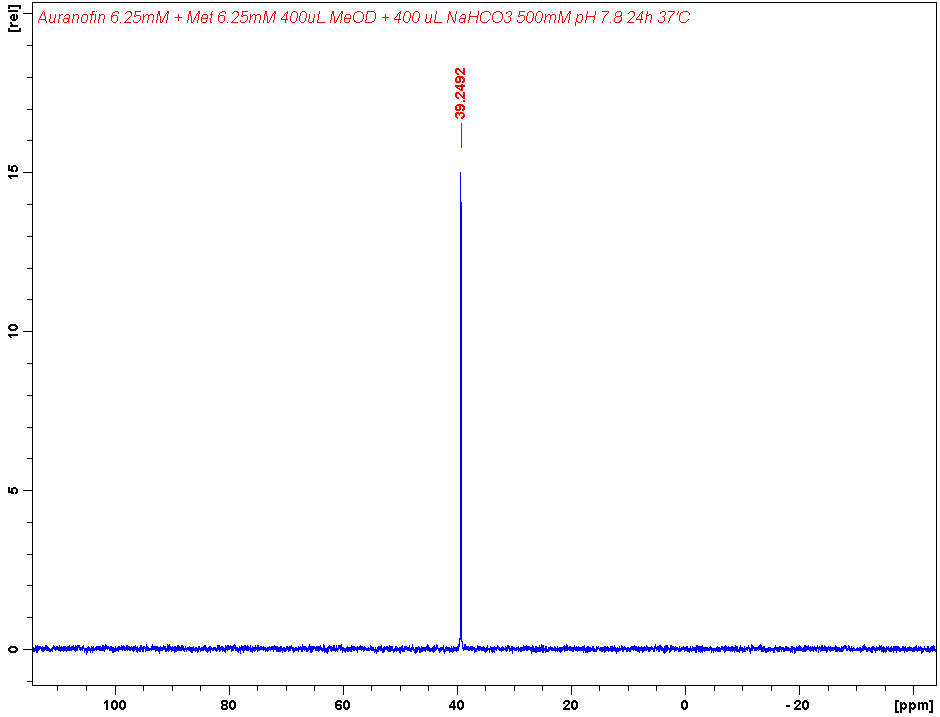


**Supplementary Figure 6.** Auranofin 6.25 mM + Met 6.25 mM in MeOD-d_4_/Carbonate buffer (500 mM pH 7.8) 1:1. Spectrum recorded after 24h of incubation at 37 °C.


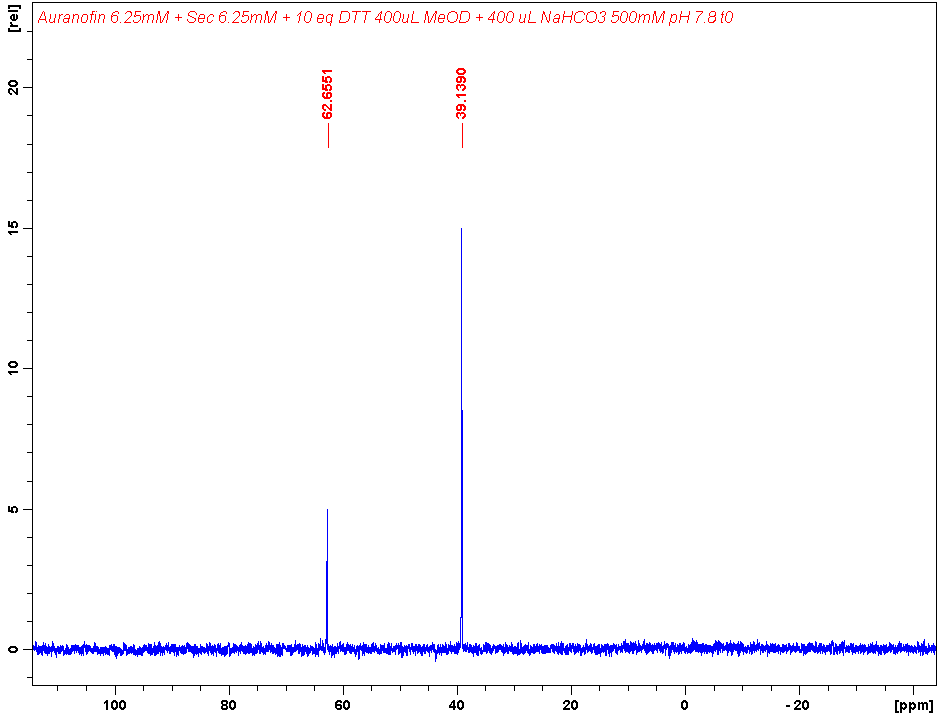


**Supplementary Figure 7.** Auranofin 6.25 mM + Sec 6.25 mM in MeOD-d_4_/Carbonate buffer (500 mM pH 7.8) 1:1. Spectrum recorded at t_0_. Sec was prepared with the addition of 10 equivalents of 1,4-Dithiothreitol to a 6.25 mM solution of selenocystine in 400 μL of carbonate buffer (500 mM pH 7.8) and subsequent incubation for 30 min at 37 °C. t_0_ is considered from the addition of Auranofin as 400 μL of a 12.5 mM solution in MeOD-d_4_.


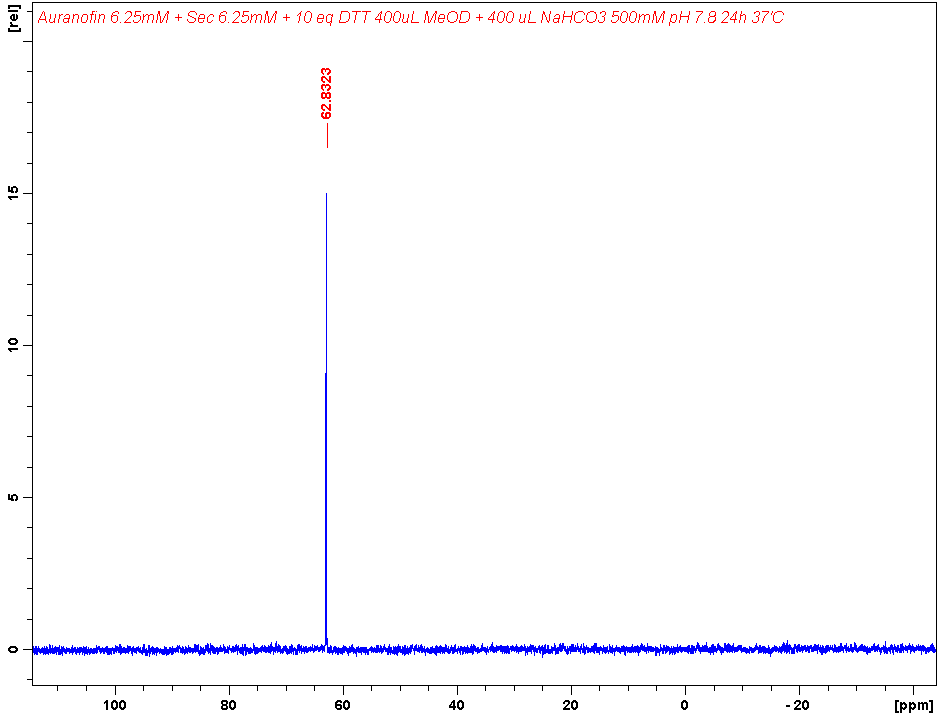


**Supplementary Figure 8.** Auranofin 6.25 mM + Sec 6.25 mM in MeOD-d_4_/Carbonate buffer (500 mM pH 7.8) 1:1. Spectrum recorded after 24h of incubation at 37 °C.


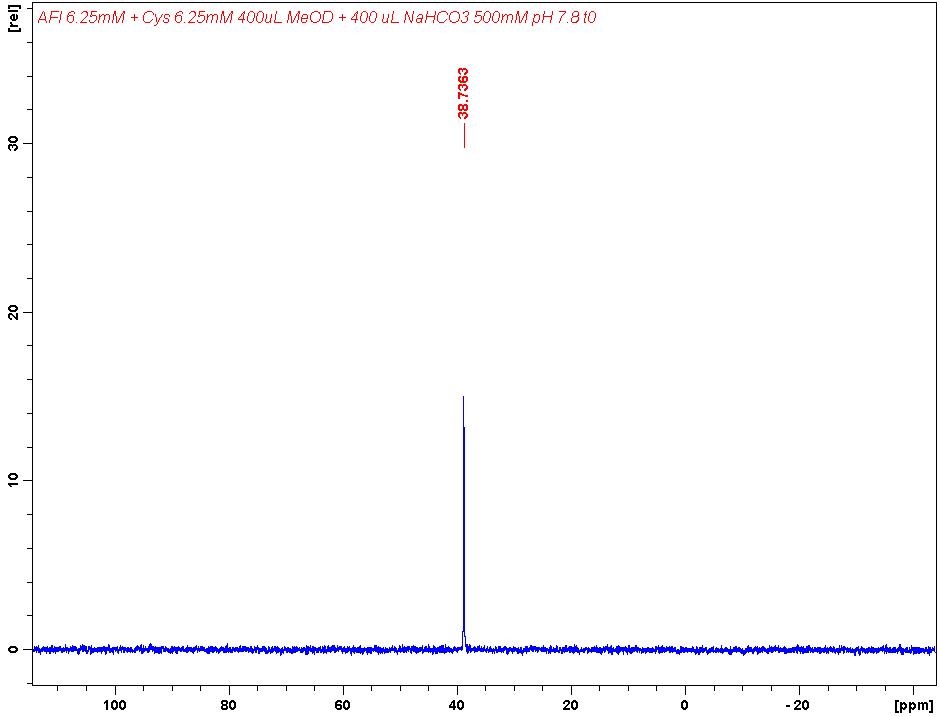


**Supplementary Figure 9.** AF-I 6.25 mM + Cys 6.25 mM in MeOD-d_4_/Carbonate buffer (500 mM pH 7.8) 1:1. Spectrum recorded at t_0_.


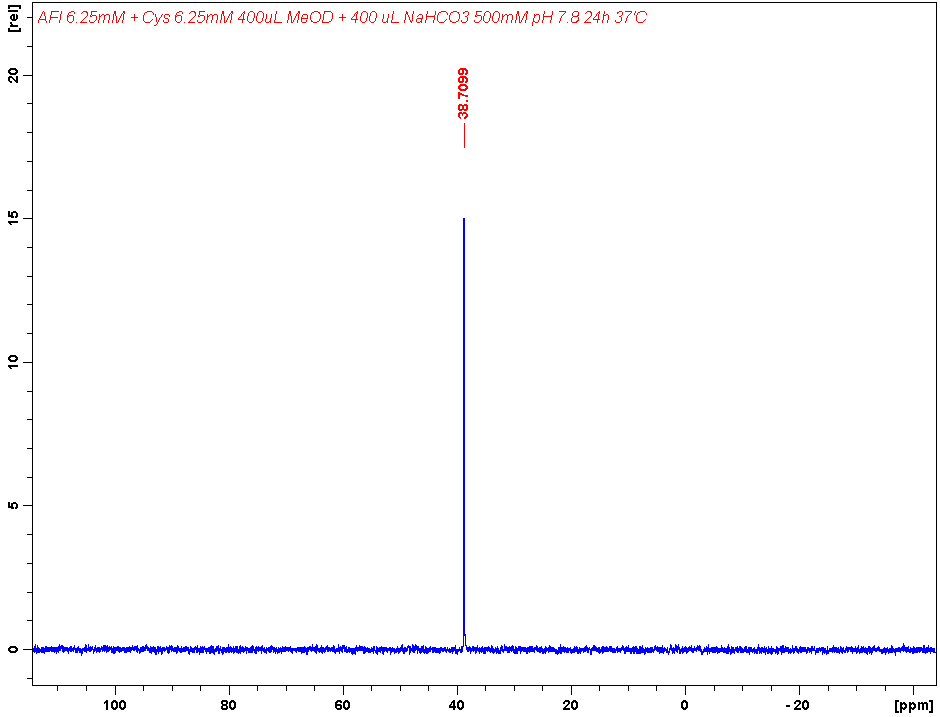


**Supplementary Figure 10.** AF-I 6.25 mM + Cys 6.25 mM in MeOD-d_4_/Carbonate buffer (500 mM pH 7.8) 1:1. Spectrum recorded after 24h of incubation at 37 °C.


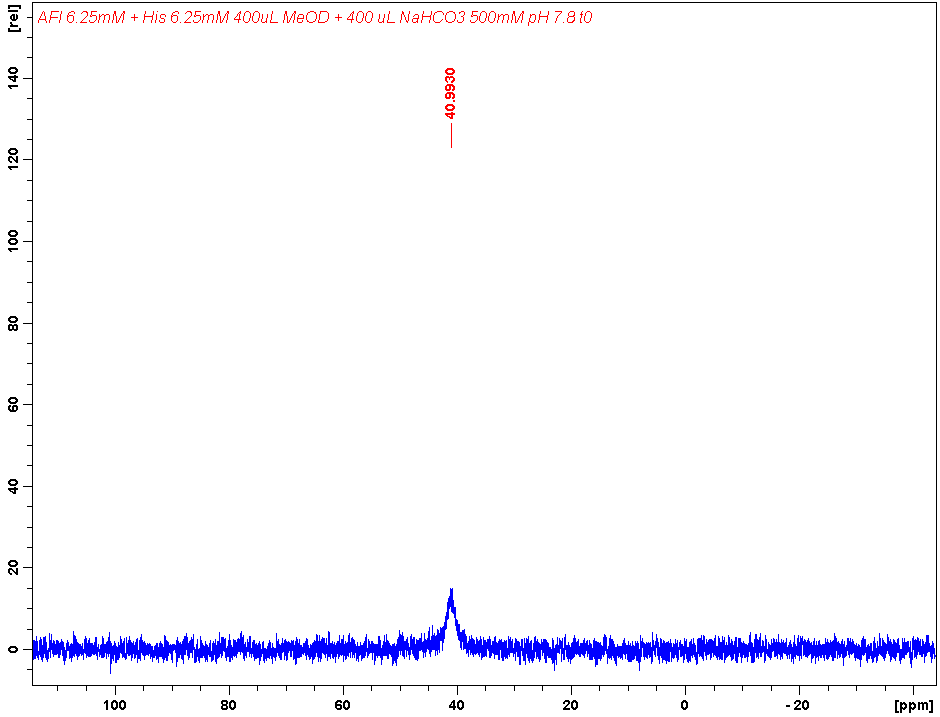


**Supplementary Figure 11.** AF-I 6.25 mM + His 6.25 mM in MeOD-d_4_/Carbonate buffer (500 mM pH 7.8) 1:1. Spectrum recorded at t_0_.


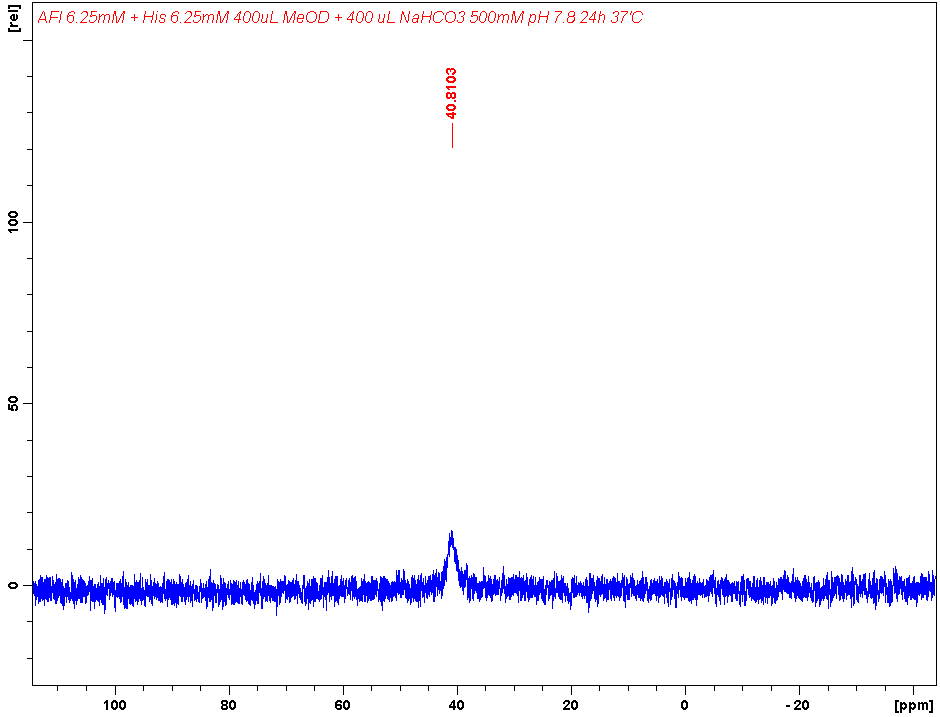


**Supplementary Figure 12.** AF-I 6.25 mM + His 6.25 mM in MeOD-d_4_/Carbonate buffer (500 mM pH 7.8) 1:1. Spectrum recorded after 24h of incubation at 37 °C.


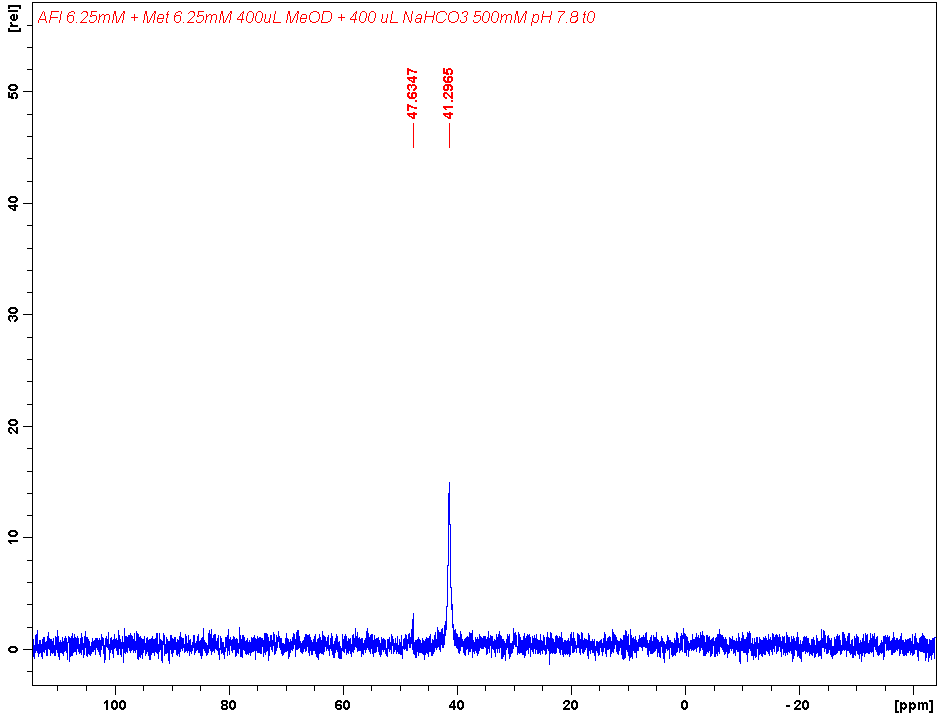


**Supplementary Figure 13.** AF-I 6.25 mM + Met 6.25 mM in MeOD-d_4_/Carbonate buffer (500 mM pH 7.8) 1:1. Spectrum recorded at t_0_.


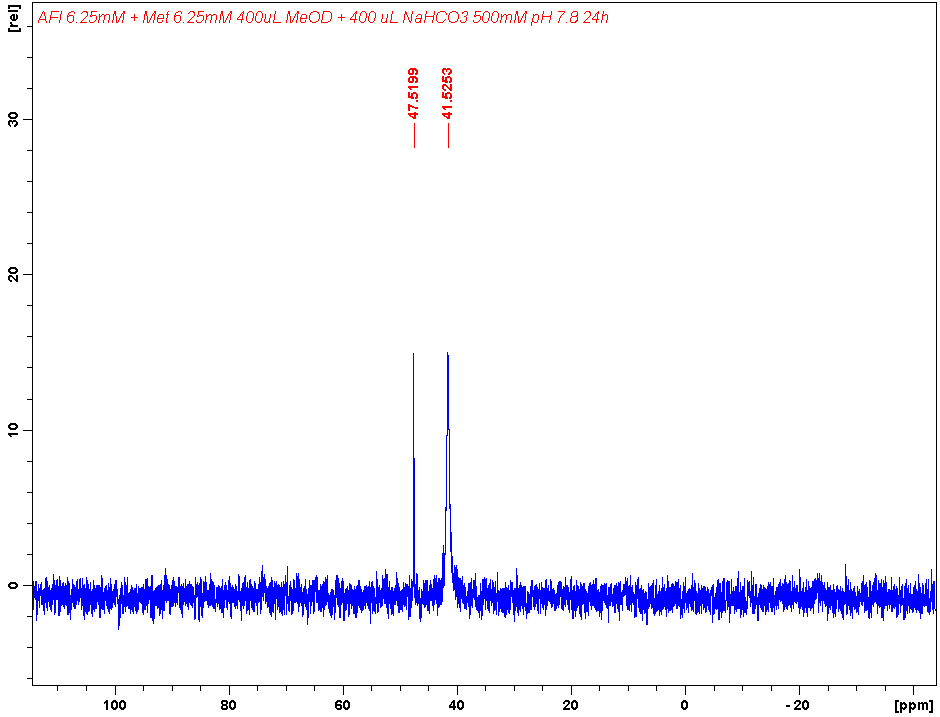


**Supplementary Figure 14.** AF-I 6.25 mM + Met 6.25 mM in MeOD-d_4_/Carbonate buffer (500 mM pH 7.8) 1:1. Spectrum recorded after 24h of incubation at 37 °C.


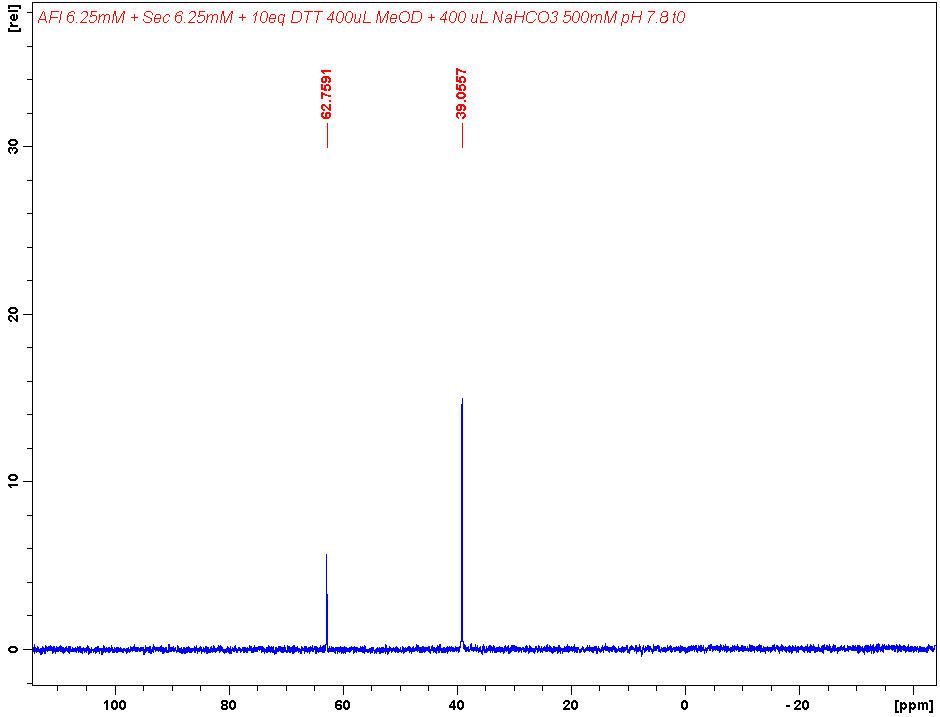


**Supplementary Figure 15.** AF-I 6.25 mM + Sec 6.25 mM in MeOD-d_4_/Carbonate buffer (500 mM pH 7.8) 1:1. Spectrum recorded at t_0_. Sec was prepared with the addition of 10 equivalents of 1,4-Dithiothreitol to a 6.25 mM solution of selenocystine in 400 μL of carbonate buffer (500 mM pH 7.8) and subsequent incubation for 30 min at 37 °C. t_0_ is considered from the addition of AF-I as 400 μL of a 12.5 mM solution in MeOD-d_4_.


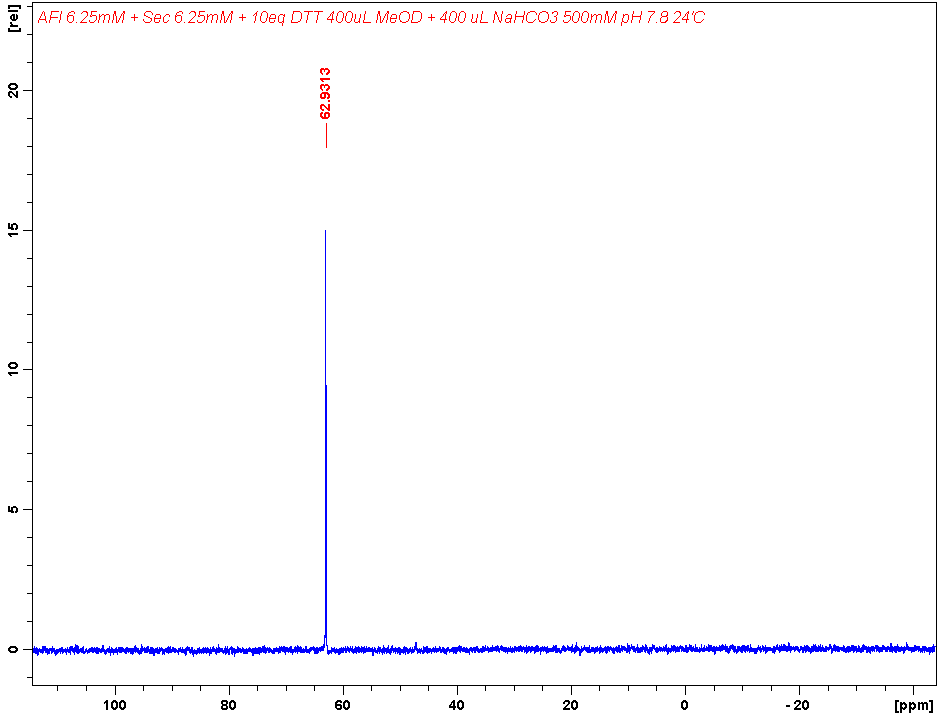


**Supplementary Figure 16.** AF-I 6.25 mM + Sec 6.25 mM in MeOD-d_4_/Carbonate buffer (500 mM pH 7.8) 1:1. Spectrum recorded after 24h of incubation at 37 °C.

# FT-IR spectra

**Supplementary Figure 17.** ATR-FTIR spectrum of Cysteine 30 mM solution in 1:1 MeOH and NaHCO_3_ buffer (500 mM), pH=7.8.

**Supplementary Figure 18.** ATR-FTIR spectrum of Auranofin 30 mM solution in 1:1 MeOH and NaHCO_3_ buffer (500 mM), pH=7.8.

**Supplementary Figure 19.** ATR-FTIR spectrum of Au(PEt_3_)I 30 mM solution in 1:1 MeOH and NaHCO_3_ buffer (500 mM), pH=7.8.

**Supplementary Figure 20.** ATR-FTIR spectrum of Cysteine 30 mM + Auranofin 30 mM solution in 1:1 MeOH and NaHCO_3_ buffer (500 mM), pH=7.8.

**Supplementary Figure 21.** ATR-FTIR spectrum of Cysteine 30 mM + Au(PEt_3_)I 30 mM solution in 1:1 MeOH and NaHCO_3_ buffer (500 mM), pH=7.8.

# ESI-MS spectra


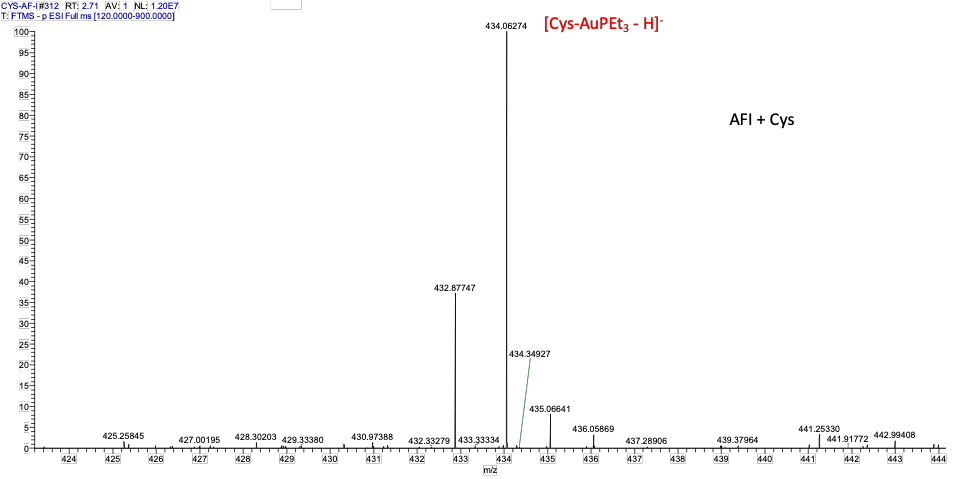


**Supplementary Figure 22.** ESI mass spectrum of AF-I (10^-5^ M) incubated for 24 h at 37 °C with cysteine (1:1 metal to amino acid ratio), in ammonium acetate buffer 500 mM pH=6.8 in presence of 50% MeOH.

**Supplementary Figure 23.** ESI mass spectrum of AF (10^-5^ M) incubated for 24 h at 37 °C with selenocysteine (1:1 metal to amino acid ratio), in ammonium acetate buffer 500 mM pH=6.8 in presence of 50% MeOH and 10 eq. of DTT.

**Supplementary Figure 24.** ESI mass spectrum of AF-I (10^-5^ M) incubated for 24 h at 37 °C with selenocysteine (1:1 metal to amino acid ratio), in ammonium acetate buffer 500 mM pH=6.8 in presence of 50% MeOH and 10 eq. of DTT.

**Supplementary Figure 25.** ESI mass spectrum of AF (10^-5^ M) incubated for 24 h at 37 °C with histidine (1:1 metal to amino acid ratio), in ammonium acetate buffer 500 mM pH=6.8 in presence of 50% MeOH.

**Supplementary Figure 26.** ESI mass spectrum of AF-I (10^-5^ M) incubated for 24 h at 37 °C with histidine (1:1 metal to amino acid ratio), in ammonium acetate buffer 500 mM pH=6.8 in presence of 50% MeOH.

**Supplementary Figure 27.** ESI mass spectrum of AF (10^-5^ M) incubated for 24 h at 37 °C with methionine (1:1 metal to amino acid ratio), in ammonium acetate buffer 500 mM pH=6.8 in presence of 50% MeOH.

**Supplementary Figure 28.** ESI mass spectrum of AF-I (10^-5^ M) incubated for 24 h at 37 °C with methionine (1:1 metal to amino acid ratio), in ammonium acetate buffer 500 mM pH=6.8 in presence of 50% MeOH.
